# ARF-BP1 as a potential therapeutic target

**DOI:** 10.1038/sj.bjc.6603119

**Published:** 2006-04-25

**Authors:** D Chen, C L Brooks, W Gu

**Affiliations:** 1 Institute for Cancer Genetics, Department of Pathology, College of Physicians & Surgeons, Columbia University, 1150 St Nicholas Ave, New York, NY 10032, USA

**Keywords:** ARF-BP1, p53, ARF, Mdm2, therapeutic

## Abstract

In this review, we discuss the recent identification of ARF-BP1 (also known as Mule, UREB1, E3^histone^, LASU1, and HectH9). ARF-BP1, a HECT domain-containing E3 ubiquitin ligase, interacts with ARF and p53. Its ubiquitin ligase activity is inhibited by ARF. Inactivation of ARF-BP1 stabilised p53 and induced apoptosis. Notably, inactivation of ARF-BP1 also caused cell growth repression in p53-null cells and breast cancer cells with mutant p53. Thus, ARF-BP1 emerges as a novel therapeutic target against cancer regardless of p53 status.

The function of p53 as a transcription factor in the induction of cell growth arrest and apoptosis has been widely known and the importance of its role in tumour suppression has been firmly established. The activity of p53 as a sequence-specific transcription factor is highly regulated by other proteins through post-translational modifications, protein–protein interactions and protein stabilisation (Brooks and Gu, 2003; Harris and Levine, 2005). Therefore, study of regulators of p53 becomes very important in exploring cancer therapy (Bykov and Wiman, 2003; Vassilev *et al*, 2004).

Until recently, Mdm2, a RING finger E3 ubiquitin ligase, was thought to be the main regulator of p53 by ubiquitination and subsequent degradation by the 26S proteasome (Oren, 2003; Vousden and Prives, 2005). ARF (known as p14^ARF^ in humans and p19^ARF^ in mouse) was originally identified as an alternative transcript of the Ink4a/ARF tumour-suppressor locus (Sherr, 2004). ARF exhibits tumour-suppressor functions, as demonstrated by the tumour susceptibility phenotype of p19^ARF^-deficient mice (Kamijo *et al*, 1997). The N-terminus region of ARF (N-ARF: residues 1–64), encoded by the unique 1*β* exon, is critical for ARF-mediated p53 activation as well as p53-independent ARF functions. The C-terminal region (C-ARF: residues 65–132), which is not conserved between human and mouse counterparts, is of uncertain function. ARF directly binds to and inactivates Mdm2 resulting in p53 stabilisation and p53-dependent apoptosis or cell cycle arrest (Quelle *et al*, 1995; Zhang *et al*, 1998). The ARF-Mdm2-p53 axis is undoubtedly an important component of ARF's tumour-suppressor activity. However, a number of recent studies have demonstrated that ARF also possesses p53- and Mdm2- independent functions. For example, ARF can induce cell growth arrest in tumour cells that lack a functional *p53* gene or its key transcriptional target *p21* (Weber *et al*, 2000; Modestou *et al*, 2001; Korgaonkar *et al*, 2002; Eymin *et al*, 2003; Normand *et al*, 2005). Expression of ARF inhibits the proliferation of cells that are null for both p53 and Mdm2 (Weber *et al*, 2000). Moreover, mice lacking both *ARF* and *p53* develop multiple primary tumours of a wider spectrum than those mice lacking either gene alone. In the same study, mice lacking *p53*, *Mdm2*, *ARF* were found to develop tumours at a higher frequency and of wider spectrum than those with mice lacking *p53* and *Mdm2* (Weber *et al*, 2000). Inactivation of ARF by hypermethylation was observed in human colorectal tumours having inactivated p53 (Esteller *et al*, 2001). Hence, ARF targets other proteins in addition to Mdm2. Recently, it has been reported that ARF interacts with B23 (nucleophosmin), a nucleolar protein implicated in ribosomal biogenesis (Bertwistle *et al*, 2004). Moreover, ARF has been shown to associate with c-Myc and inhibit the expression of c-Myc-activated genes (Qi *et al*, 2004). Interestingly, ARF interacts with certain members of the E2F family of transcription factors (Martelli *et al*, 2001). All of these might in part explain the p53-independent function of ARF, but the mechanisms remain unclear.

## ARF-BP1, A HECT E3 UBIQUITIN LIGASE, PLAYS AN IMPORTANT ROLE IN THE MEDIATION OF P53 DEPENDENT AND INDEPENDENT EFFECTS ON ARF

In a recent study, a major protein band of ∼500 kDa (named ARF-BP1) was purified from HA-ARF-Flag-expressing H1299 cells by mass spectrometric analysis (Chen *et al*, 2005). The C-terminal sequences of ARF-BP1 (also known as Mule, UREB1, E3^histone^, LASU1 and HectH9) possess a HECT domain (4036–4374) (Adhikary *et al*, 2005; Chen *et al*, 2005; Liu *et al*, 2005; Zhong *et al*, 2005). ARF-BP1 also contains the ubiquitin-associated domain (UBA, 1318–54) and a WWE domain (1612–92). The former is a small sequence motif found in various proteins linked to the ubiquitination pathway such as the DNA repair protein Rad23 and the Cbl ubiquitin ligase. The latter may be involved in protein–protein interactions. ARF-BP1 mRNA is broadly expressed in various types of human tissue (Chen *et al*, 2005).

A region of ARF-BP1 (1015–4574) not including the N-terminus (1–1014) strongly bound the N-terminal domain of ARF. ARF and ARF-BP1 also interact with each other under physiological conditions as shown by co-immunoprecipitations of H1299 cell lysates with *α*-ARF-BP1 or *α*-ARF (Chen *et al*, 2005). ARF-BP1 displays E3 enzymatic activity in an *in vitro* assay, and this self-ubiquitination activity was strongly repressed by ARF (Chen *et al*, 2005). Notably, ARF-BP1 inactivation by RNAi induces cell growth repression with G2/M arrest in p53-null cells H1299 and Saos-2, similar to that seen in response to ARF induction (Eymin *et al*, 2003; Chen *et al*, 2005; Normand *et al*, 2005). Based on our observation that ARF-BP1 is the major binding partner of ARF in p53-null cells, the ability of ARF to neutralise ARF-BP1 plays a critical contribution to the p53-independent antiproliferative effects of ARF (Chen *et al*, 2005; Shmueli and Oren, 2005). Future work is necessary to clarify the precise mechanism of ARF-BP1-mediated and p53-independent cell growth inhibition induced by ARF. As ARF-BP1 is a bona fide E3 ubiquitin ligase, it is likely that the p53-independent functions of ARF involve unidentified enzymatic substrates of ARF-BP1.

Surprisingly, ARF-BP1 can interact directly with the p53 protein and induces p53 ubiquitination. Significantly, ARF-BP1-mediated p53 ubiquitination was strongly repressed by ARF (Chen *et al*, 2005). Also, the N-terminal region of ARF retained full inhibition of ARF-BP1-mediated p53 ubiquitination whereas the C-terminal region showed no effect. This is consistent with the previous reports that N-terminal ARF has full functional responses (Weber *et al*, 1999; Midgley *et al*, 2000). RNAi-mediated knockdown of ARF-BP1 stabilised p53 and induced apoptosis (32.3%) in human osteosarcoma cells (U2OS). Similar results were achieved using different cell lines with wild-type p53, such as human breast carcinoma cells (MCF-7), human lung adenocarcinoma cells (A549) and normal human fibroblast cells (NHF-1) (Chen *et al*, 2005). An inverse correlation between ARF-BP1 and p53 expression in colorectal carcinoma samples has also been shown by immunofluorescence (Yoon *et al*, 2005). By overexpression, ARF can stabilise p53 in *p53/Mdm2* double-null MEF cells. Moreover, the p53 stabilisation induced by ARF was clearly attenuated by further ARF-BP1 knockdown of these cells, suggesting that ARF-BP1 is critical for ARF-mediated p53 stabilisation in an Mdm2-independent manner (Chen *et al*, 2005).

In addition, two other recently identified proteins, Pirh2 and COP1, are also found to promote p53 degradation via the ubiquitin-proteasome pathway (Leng *et al*, 2003; Corcoran *et al*, 2004; Dornan *et al*, 2004). We tested the differential effects of the known E3 ligases on p53 stabilisation by RNAi knockdown (Chen *et al*, 2005). Depletion of Mdm2 stabilised p53 while depletion of either COP1 or Pirh2 had a modest effect. Remarkably, inactivation of ARF-BP1 strongly induced p53 stabilisation and activated p53-mediated transcription; among the known E3 ligases for p53, ARF-BP1 RNAi induced the highest levels of p21 and Bax, and exhibited similar effects to ARF overexpression. Hence, ARF-BP1 is a major E3 ubiquitin ligase for p53, and more importantly, is also a key mediator for ARF tumour-suppressor function.

It is now clear that ARF interacts with and regulates at least two distinct E3 ubiquitin ligases, Mdm2 and ARF-BP1, for their ubiquitination of p53 (den Besten *et al*, 2005). This is consistent with the previous evidence suggesting that ARF-mediated activation of p53 is more complicated than the simple ARF-Mdm2-p53 model. First, while the low levels of Mdm2 that are commonly observed in normal cells preferentially catalyse monoubiquitination of p53 (Li *et al*, 2003), ARF can not inhibit Mdm2-mediated monoubiquitination of p53 *in vivo*, although it can block p53 polyubiquitination (Xirodimas *et al*, 2001). Second, recent studies of p19^ARF^ demonstrated that ARF is able to cause cell cycle arrest independent of Mdm2 relocalisation to nucleoli and p53 stabilisation (Llanos *et al*, 2001; Korgaonkar *et al*, 2002). Thus, a critical question raised is how ARF can efficiently stabilise p53 in normal cells with low levels of Mdm2, a situation where Mdm2 is not solely responsible for p53 degradation. Our results with ARF-BP1 suggest a novel pathway independent of Mdm2 for ARF-mediated p53 activation. ARF-BP1 was identified as a significant component of ARF-containing protein complexes and endogenous protein interaction between ARF-BP1 and ARF is easily detected in unstressed cells. These two distinct pathways for ARF-mediated p53 activation allow for more precise control of p53, but also raise the question of their biological significance. For example, it is well established that Mdm2 plays a critical role in tumorigenesis. Protein overexpression and gene amplification of Mdm2 are found in various types of tumours (Michael and Oren, 2003). Thus, the ARF-Mdm2 pathway might be especially important in cells expressing high levels of Mdm2. Interestingly, we found that ARF-BP1 is highly expressed in 80% (16 out of 20) of breast cancer cell lines while the expression level of ARF-BP1 in normal breast cells (MCF-10A) is low, suggesting a potential role of ARF-BP1 in breast cancer tumorigenesis.

**Figure 1:**
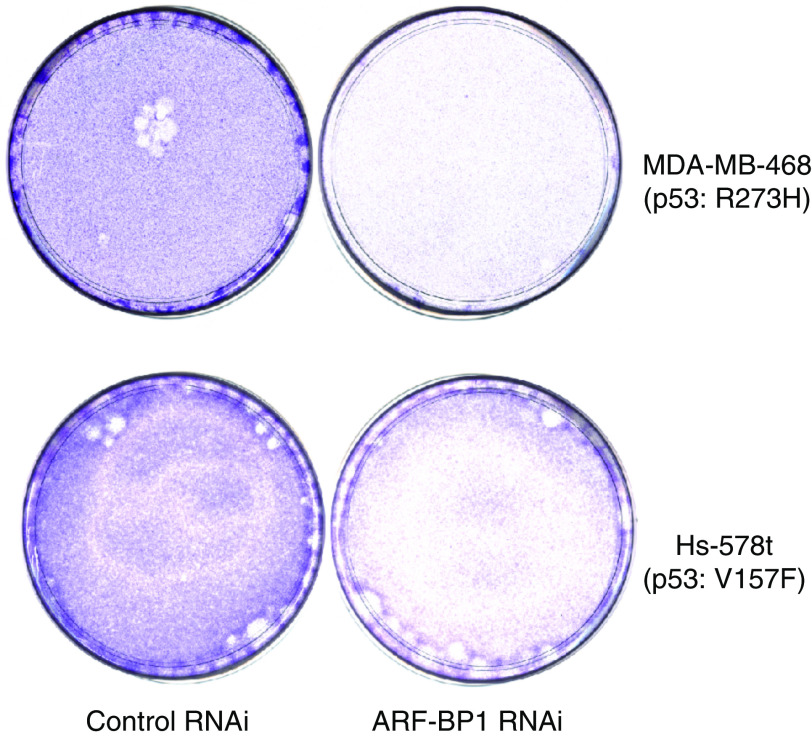
Inactivation of ARF-BP1 induces cell growth repression of breast cancer cell lines (MDA-MB-468, Hs-578t) with mutant p53. The breast cancer cells (MDA-MB-468 or Hs-578t) were stained with crystal violet 3 days after three rounds treatment with either control or ARF-BP1 RNAi.

The importance of p53 in cancer development is illustrated by the fact that p53 is highly mutated in many different cancers. The mutations are found mainly in the specific DNA-binding core domain of p53-residues 98–292 (Soussi and Lozano, 2005). It may be interesting to deduce the functional consequence of ARF-BP1 reduction in cells expressing mutant p53. Significantly, inactivation of ARF-BP1 could also inhibit cell growth in cancer cells with mutant p53 (MDA-MB-468, Hs-578t) (Figure 1). Similar results were also found in other breast cancer cells with mutant p53 such as T47D, BT-549, MDA-MB-435, SK-BR-3.

## ADDITIONAL TARGETS OF ARF-BP1/HectH9

**Figure 2:**
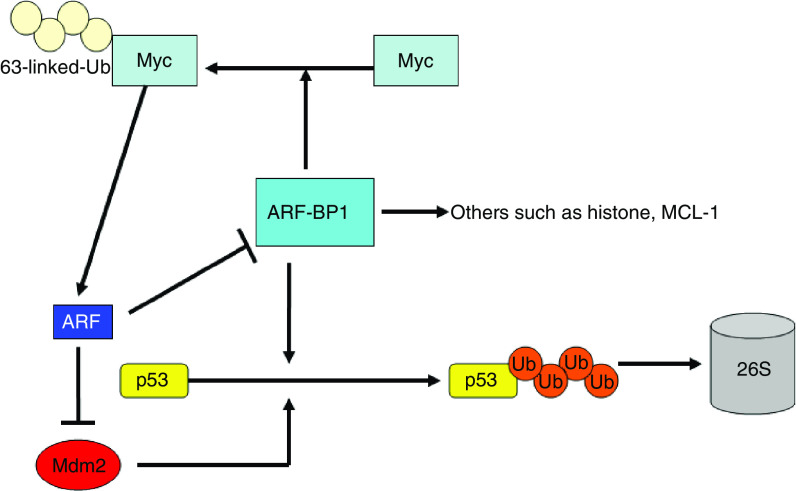
The role of ARF-BP1. The important role of ARF-BP1 as a major ubiquitin ligase for p53 in an Mdm2-independent pathway. ARF-BP1 also functions as an E3 ubiquitin ligase for Myc and regulates the switch between the activated and repressed state of Myc protein. ARF-BP1 may have other substrates such as histones and Mcl-1. Induction of ARF by Myc resulting in inhibition of ARF-BP1 may serve as a negative feedback loop to limit excessive Myc functions.

It was recently reported that ARF-BP1 ubiquitinated Myc through a lysine 63-linked polyubiquitin chain (Adhikary *et al*, 2005). This ubiquitation does not cause Myc degradation (Adhikary *et al*, 2005), consistent with the absence of Myc stabilisation when ARF-BP1 is inactivated (Chen *et al*, 2005). However, the ubiquitination of Myc by ARF-BP1 significantly alters transcription properties of Myc. Depletion of ARF-BP1 downregulates Myc-dependent gene activation and represses cell proliferation induced by Myc. Similarly, depletion of ARF-BP1 by RNAi leads to growth arrest in HeLa cells, despite a high endogenous level of Myc expression (Adhikary *et al*, 2005). Thus, it appears that ARF-BP1 functionally links Myc and ARF. ARF, ARF-BP1, and Myc can form a ternary complex since ARF and Myc bind each other directly (Qi *et al*, 2004). It is reasonable that the inhibition of ARF-BP1 by ARF could contribute at least in part to ARF-mediated inhibition of Myc transcriptional activation, although it has not been established yet that ARF inhibits Myc ubiquitination mediated by ARF-BP1. Myc seems to be one of the ARF-BP1 p53-independent substrates. The induction of ARF by Myc may serve as a negative feedback loop to limit excessive Myc function (Figure 2). High expression of ARF-BP1 may disrupt this feedback loop and is essential for tumorigenesis. ARF-BP1/HectH9 is indeed overexpressed in a variety of primary tumour samples (Adhikary *et al*, 2005; Yoon *et al*, 2005). In addition, ARF-BP1/Mule recently was reported to ubiquitinate and degrade the anti-apoptotic protein Mcl-1 (Warr *et al*, 2005; Zhong *et al*, 2005). The physiologic consequences of degrading both proapoptotic and antiapoptotic proteins remain unclear, although it is possible that ARF-BP1 differentially targets these proteins under different states of cell stress and/or other physiologic conditions. These other targets may somehow limit the therapeutic potential of ARF-BP1 in cancer therapy. In the future, it will be important to ascertain specific circumstances in which proteins like MCl-1 are activated, so that these situations can be avoided in treatments targeting the ARF-BP1-p53/myc pathway. Moreover, future work is necessary to uncover additional molecular substrates of ARF-BP1 as suggested by its large size (Shmueli and Oren, 2005).

## ARF-BP1 AS A THERAPEUTIC POTENTIAL FOR CANCER

Recently, numerous studies indicate that activating p53 is very important for the function of many cancer therapy drugs *in vivo*; p53-dependent apoptosis seems to have a major role for the efficiency of cancer chemotherapy in cancer cells expressing wild type p53. The negative regulation of p53 by ARF-BP1 has now been established (Gu *et al*, 1995; Chen *et al*, 2005). Based on our results that inactivation of ARF-BP1 expression activates p53 function, ARF-BP1-mediated negative regulation of p53 might be a very important target for cancer therapy. Thus, potential drugs that can downregulate ARF-BP1 protein levels or abrogate the ARF-BP1-p53 interaction *in vivo* may sensitise tumour cells. Similarly, agents that activate the p53 tumour-suppression pathway, including Nutlin and HLI98, small molecules that block the p53-Mdm2 interaction (Vassilev *et al*, 2004), and inhibit the E3 ubiquitin ligase activity of Mdm2, respectively, (Yang *et al*, 2005) are currently being tested for their potential use in cancer therapy. However, the utility of these agents is limited to tumours that maintain a functional p53 pathway, a significant restriction given that p53 is often inactivated by mutations. Since inactivation of ARF-BP1 could also induce growth arrest in p53-null or mutant p53 cells, at least partially through downregulation of Myc-target genes, inhibitors of ARF-BP1 should suppress tumour cell growth in a wide spectrum of tumour types. Therefore, ARF-BP1 may prove to be an especially valuable therapeutic target for a wider variety of cancer types.
